# Supplementary material for: Gulls in Porto Coastline as Reservoirs for Salmonella spp.: Findings from 2008 and 2023
Source: Microorganisms. 2023 Dec 28;12(1):59. doi: 10.3390/microorganisms12010059 (PMC10819206; doi:10.3390/microorganisms12010059)
Supplement: Supplementary file 1 [file microorganisms-12-00059-s001.zip › microorganisms-2764623-supplementary.pdf]

Supplementary Materials

Table S1. Accession numbers for the selected *Salmonella* isolates**.**

Isolate	Accession number
PT_SE0336	ERR12313960
PT_SE0337	ERR12313960
PT_SE0241	ERR10368852
PT_SE0173	ERR10368815
PT_SE0338	ERR12313942
PT_SE0242	ERR10368862
PT_SE0248	ERR10368802
PT_SE0243	ERR10368841
PT_SE0339	ERR12313920
PT_SE0247	ERR10368809
PT_SE0244	ERR10368811
PT_SE0245	ERR10368826
PT_SE0340	ERR12313958
PT_SE0341	ERR12313959
PT_SE0342	ERR12313943
PT_SE0246	ERR10368804
PT_SE0343	ERR12313952
PT_SE0344	ERR12313949
PT_SE0345	ERR12313925
PT_SE0174	ERR10368855
PT_SE0346	ERR12313944
PT_SE0347	ERR12313954
PT_SE0171	ERR10368796
PT_SE0348	ERR12313933
PT_SE0349	ERR12313940
PT_SE0350	ERR12313938
PT_SE0351	ERR12313951
PT_SE0352	ERR12313957
PT_SE0353	ERR12313919
PT_SE0354	ERR12313936
PT_SE0355	ERR12313948
PT_SE0356	ERR12313923
PT_SE0357	ERR12313941

Table S2. Fisher's Exact Test comparing antibiotic resistance between feces from 2008 and 2023.

Antibiotic Resistance Profile	Fisher's Exact Test (p)
AMP	0.085
CFZ	0.640
CHL	1.000
DOX	0.346
GEN	1.000
STR	0.137
SXT	0.348
TET	**0.018**
TOB	1.000

AMC: amoxicillin-clavulanate; CFZ: cefazoline; DOX: doxycycline; GEN: gentamycin; STR: streptomycin; SXT: sulfamethoxazole-trimethoprim; TET: tetracycline; TOB: tobramycin.

Table S3. Fisher's Exact Test comparing antibiotic resistance between feces and water samples from 2023.

Antibiotic Resistance Profile	Fisher's Exact Test (p)
AMP	0.367
DOX	0.367
STR	0.096
SXT	0.137
TET	0.519

AMC: amoxicillin-clavulanate; DOX: doxycycline; STR: streptomycin; SXT: sulfamethoxazole-trimethoprim; TET: tetracycline.

Table S4. Summary of phenotypic resistance and resistance genes by antibiotic class of the selected isolates from 2008.

Isolate	Aminoglycosides		Carbapenems		Cephalosporins		Folate inhibitors		Monobactams		Nitrofurans		Penicillins		Phenicols		Quinolones		Tetracyclines		Polymyxins		Genome
	R	S	R	S	R	S	R	S	R	S	R	S	R	S	R	S	R	S	R	S	R	S	
PT_SE0336	1/4	3/4	0/1	1/1	0/4	4/4	0/1	1/1	0/1	1/1	0/1	1/1	0/2	2/2	0/1	1/1	0/2	2/2	2/2	0/2	0/1	1/1	*aac(6′)-Iaa*, *fosA7*, *tet(B)*, *parC*:T57S
PT_SE0337	0/4	4/4	0/1	1/1	0/4	4/4	0/1	1/1	0/1	1/1	0/1	1/1	0/2	2/2	0/1	1/1	0/2	2/2	0/2	2/2	0/1	1/1	*aac(6′)-Iaa*, *parC:p.T57S*
PT_SE0241	1/4	3/4	0/1	1/1	0/4	4/4	0/1	1/1	0/1	1/1	0/1	1/1	0/2	2/2	0/1	1/1	0/2	2/2	0/2	2/2	0/1	1/1	*aac(6′)-Iaa*
PT_SE0173	1/4	3/4	0/1	1/1	0/4	4/4	0/1	1/1	0/1	1/1	0/1	1/1	0/2	2/2	0/1	1/1	0/2	2/2	2/2	0/2	0/1	1/1	*aac(6′)-Iaa*, *fosA7*, *tet(B)*, *parC*:T57S
PT_SE0338	0/4	4/4	0/1	1/1	0/4	4/4	0/1	1/1	0/1	1/1	0/1	1/1	0/2	2/2	0/1	1/1	0/2	2/2	0/2	2/2	0/1	1/1	*aac(6′)-Iaa*, *aac(2′)-IIa*, *parC*:T57S
PT_SE0242	1/4	3/4	0/1	1/1	0/4	4/4	0/1	1/1	0/1	1/1	0/1	1/1	1/2	1/2	0/1	1/1	0/2	2/2	0/2	2/2	0/1	1/1	*aac(6′)-laa*, *aph(3′′)-Ib*, *aph(6)-Id*, *bla*$_{TEM-1B}$, *sul2*
PT_SE0248	0/4	4/4	0/1	1/1	0/4	4/4	0/1	1/1	0/1	1/1	0/1	1/1	0/2	2/2	0/1	1/1	0/2	2/2	0/2	2/2	0/1	1/1	*aac(6′)-Iaa*
PT_SE0243	1/4	3/4	0/1	1/1	1/4	3/4	1/1	0/1	0/1	1/1	0/1	1/1	1/2	1/2	0/1	1/1	0/2	2/2	2/2	0/2	0/1	1/1	*aac(6′)-Iaa*, *aph(3′′)-Ib*, *aph(6)-Id*, *bla*$_{TEM-1B}$, *dfrA14*, *sul2*, *tet(A)*
PT_SE0339	1/4	3/4	0/1	1/1	0/4	4/4	0/1	1/1	0/1	1/1	0/1	1/1	0/2	2/2	0/1	1/1	0/2	2/2	0/2	2/2	0/1	1/1	*aac(6′)-Iaa*, *parC*:T57S
PT_SE0247	0/4	4/4	0/1	1/1	0/4	4/4	0/1	1/1	0/1	1/1	0/1	1/1	0/2	2/2	0/1	1/1	0/2	2/2	0/2	2/2	0/1	1/1	*aac(6′)-Iaa*, *fosA7*, *parC*:T57S
PT_SE0244	1/4	3/4	0/1	1/1	1/4	3/4	1/1	0/1	0/1	1/1	0/1	1/1	1/2	1/2	0/1	1/1	0/2	2/2	2/2	0/2	0/1	1/1	*aac(6′)-Iaa*, *aph(3′′)-Ib*, *aph(6)-Id*, *bla*$_{TEM-1B}$, *dfrA14*,, *sul2*, *tet(A)*
PT_SE0245	0/4	4/4	0/1	1/1	1/4	3/4	1/1	0/1	0/1	1/1	0/1	1/1	1/2	1/2	0/1	1/1	0/2	2/2	2/2	0/2	0/1	1/1	*aac(6′)-Iaa*, *aph(3′′)-Ib*,

																						aph(6)-Id, *bla*$_{TEM-1B}$, *dfrA14*, *sul2*, *tet(A)*
PT_SE0340	0/4	4/4	0/1	1/1	0/4	4/4	1/1	0/1	0/1	1/1	0/1	1/1	1/2	1/2	0/1	1/1	0/2	2/2	2/2	0/2	0/1	1/1	*aac(6′)-Iaa*, *aph(3′′)-Ib*, *aph(6)-Id*, *bla*$_{TEM-1B}$, *dfrA14*, *sul2*, *tet(A)*
PT_SE0341	1/4	3/4	0/1	1/1	0/4	4/4	0/1	1/1	0/1	1/1	0/1	1/1	1/2	1/2	0/1	1/1	0/2	2/2	2/2	0/2	0/1	1/1	*aac(6′)-Iaa*, *aph(3′′)-Ib*, *aph(6)-Id*, *bla*$_{TEM-1B}$, *dfrA14*, *sul2*, *tet(B)*
PT_SE0342	1/4	3/4	0/1	1/1	1/4	3/4	1/1	0/1	0/1	1/1	0/1	1/1	1/2	1/2	0/1	1/1	0/2	2/2	2/2	0/2	0/1	1/1	*aac(6′)-Iaa*, *aph(3′′)-Ib*, *aph(6)-Id*, *bla*$_{TEM-1B}$, *dfrA14*, *sul2*, *tet(A)*
PT_SE0246	1/4	3/4	0/1	1/1	0/4	4/4	0/1	1/1	0/1	1/1	0/1	1/1	0/2	2/2	0/1	1/1	0/2	2/2	0/2	2/2	0/1	1/1	*aac(6′)-Iaa*
PT_SE0343	1/4	3/4	0/1	1/1	0/4	4/4	0/1	1/1	0/1	1/1	0/1	1/1	1/2	1/2	0/1	1/1	0/2	2/2			0/1	1/1	*aac(6′)-Iaa*, *aph(3′′)-Ib*, *aph(6)-Id*, *bla*$_{TEM-1B}$, *sul2*
PT_SE0344	0/4	4/4	0/1	1/1	0/4	4/4	0/1	1/1	0/1	1/1	0/1	1/1	0/2	2/2	0/1	1/1	0/2	2/2	2/2	0/2	0/1	1/1	*aac(6′)-Iaa*, *tet(A)*, *parC*:T57S
PT_SE0345	0/4	4/4	0/1	1/1	0/4	4/4	0/1	1/1	0/1	1/1	0/1	1/1	0/2	2/2	0/1	1/1	0/2	2/2	0/2	2/2	0/1	1/1	*aac(6′)-Iaa*
PT_SE0174	1/4	3/4	0/1	1/1	0/4	4/4	0/1	1/1	0/1	1/1	0/1	1/1	0/2	2/2	0/1	1/1	0/2	2/2	0/2	2/2	0/1	1/1	*aac(6′)-laa*, *fosA7*, *parC*:T57S
PT_SE0346	1/4	3/4	0/1	1/1	0/4	4/4	0/1	1/1	0/1	1/1	0/1	1/1	0/2	2/2	0/1	1/1	0/2	2/2	2/2	0/2	0/1	1/1	*aac(6′)-Iaa*, *aadA2b*, *fosA7*, *parC*:T57S, *qacE*, *sul1*, *tet(A)*
PT_SE0347	0/4	4/4	0/1	1/1	0/4	4/4	0/1	1/1	0/1	1/1	0/1	1/1	0/2	2/2	0/1	1/1	0/2	2/2	0/2	2/2	0/1	1/1	*aac(6′)-Iaa*, *parC*:T57S

PT_SE0171	0/4	4/4	0/1	1/1	0/4	4/4	0/1	1/1	0/1	1/1	0/1	1/1	0/2	2/2	0/1	1/1	0/2	2/2	0/2	2/2	0/1	1/1	*aac(6′)-Iaa*
PT_SE0348	1/4	3/4	0/1	1/1	0/4	4/4	0/1	1/1	0/1	1/1	0/1	1/1	0/2	2/2	0/1	1/1	0/2	2/2	2/2	0/2	0/1	1/1	*aac(6′)-Iaa*, *aph(3′′)-Ib*, *aph(6)-Id*, bla_{TEM-1B}, *sul2*, *tet(B)*
PT_SE0349	0/4	4/4	0/1	1/1	0/4	4/4	0/1	1/1	0/1	1/1	0/1	1/1	0/2	2/2	0/1	1/1	0/2	2/2	0/2	2/2	0/1	1/1	*aac(6′)-Iaa*, *parC*:T57S
PT_SE0350	3/4	1/4	0/1	1/1	0/4	4/4	1/1	0/1	0/1	1/1	0/1	1/1	0/2	2/2	1/1	0/1	0/2	2/2	2/2	0/2	0/1	1/1	*aac(6′)-Iaa*, *aph(3′′)-Ib*, *aph(4)-Ia*, *aph(6)-Id*, *aadA1*, *aac(3)-IV*, *floR*, *dfrA1*, *parC*:T57S, *qacE*, *sul1*, *tet(A)*

All results with intermediate susceptibility were classified as resistant.

Table S5. Summary of phenotypic resistance and resistance genes by the antibiotic class of the selected isolates from 2023.

Isolate	Aminoglycosides		Carbapenems		Cephalosporins		Folate inhibitors		Monobactams		Nitrofurans		Penicillins		Phenicols		Quinolones		Tetracyclines		Polymyxins		Genome
	R	S	R	S	R	S	R	S	R	S	R	S	R	S	R	S	R	S	R	S	R	S	
PT_SE0351	1/4	3/4	0/1	1/1	0/4	4/4	0/1	1/1	0/1	1/1	0/1	1/1	1/2	1/2	0/1	1/1	0/2	2/2	0/2	2/2	0/1	1/1	*aac(6′)-Iaa*, *aph(6)-Id*, *aph(3′′)-Ib*, bla_{TEM-1B}, *sul2*
PT_SE0352	0/4	4/4	0/1	1/1	0/4	4/4	0/1	1/1	0/1	1/1	0/1	1/1	0/2	2/2	0/1	1/1	0/2	2/2	0/2	2/2	0/1	1/1	*aac(6′)-Iaa*, *parC*:T57S
PT_SE0353	1/4	3/4	0/1	1/1	0/4	4/4	0/1	1/1	0/1	1/1	0/1	1/1	1/2	1/2	0/1	1/1	0/2	2/2	2/2	0/2	0/1	1/1	*aac(6′)-Iaa*, *aph(6)-Id*, *aph(3′′)-Ib*, bla_{TEM-1B}, *sul2*, *tet(B)*
PT_SE0354	1/4	3/4	0/1	1/1	0/4	4/4	1/1	0/1	0/1	1/1	0/1	1/1	1/2	1/2	0/1	1/1	0/2	2/2	2/2	0/2	0/1	1/1	*aac(6′)-Iaa*, *aadA1*, *aadA2*, bla_{TEM-1B}, *drfA12*,

																							mph(A), *parC*:T57S, *qacE* , *sul1*, *tet(A)*
PT_SE0355	1/4	3/4	0/1	1/1	0/4	4/4	0/1	1/1	0/1	1/1	0/1	1/1	0/2	2/2	0/1	1/1	0/2	2/2	0/2	2/2	0/1	1/1	*aac(6′)-Iaa*, *parC*:T57S
PT_SE0356	1/4	3/4	0/1	1/1	0/4	4/4	0/1	1/1	0/1	1/1	0/1	1/1	0/2	2/2	0/1	1/1	0/2	2/2	0/2	2/2	0/1	1/1	*aac(6′)-Iaa*
PT_SE0357	1/4	3/4	0/1	1/1	0/4	4/4	0/1	1/1	0/1	1/1	0/1	1/1	0/2	2/2	0/1	1/1	0/2	2/2	0/2	2/2	0/1	1/1	*aac(6′)-Iaa*

All results with intermediate susceptibility were classified as resistant.

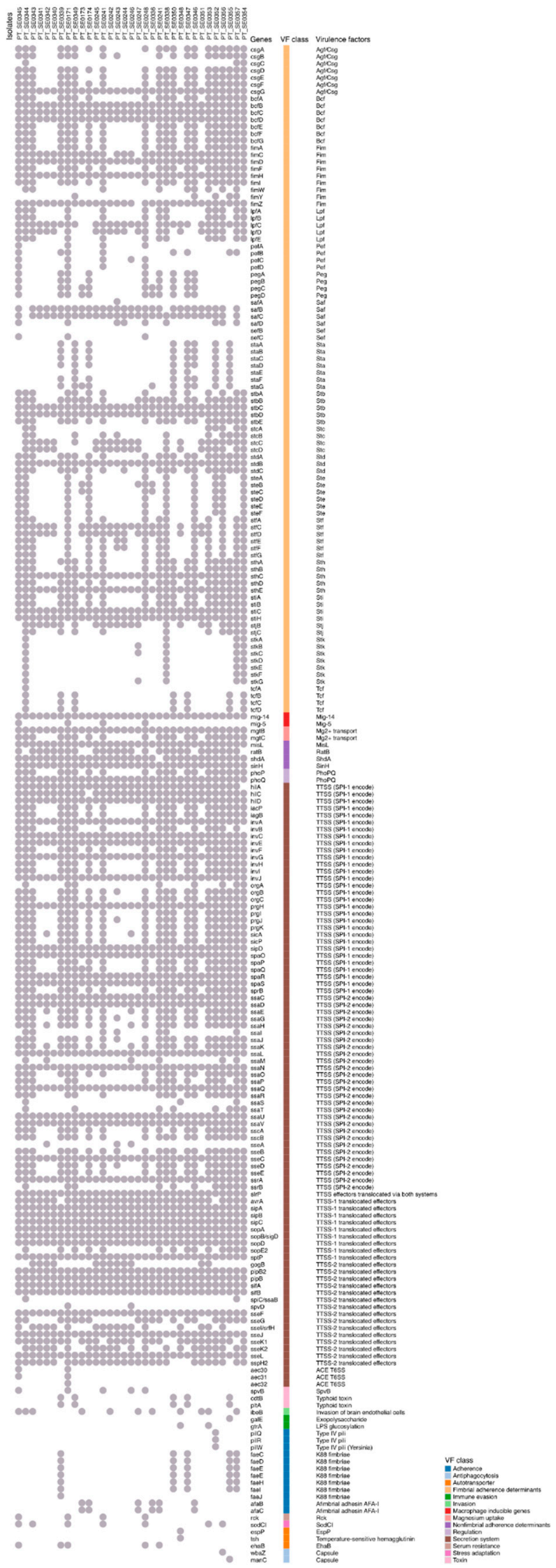

Figure S1. Heatmap illustrating the virulence factor (VF) classes identified in the genome of the 33 selected *Salmonella* isolates from 2008 and 2023 collections.

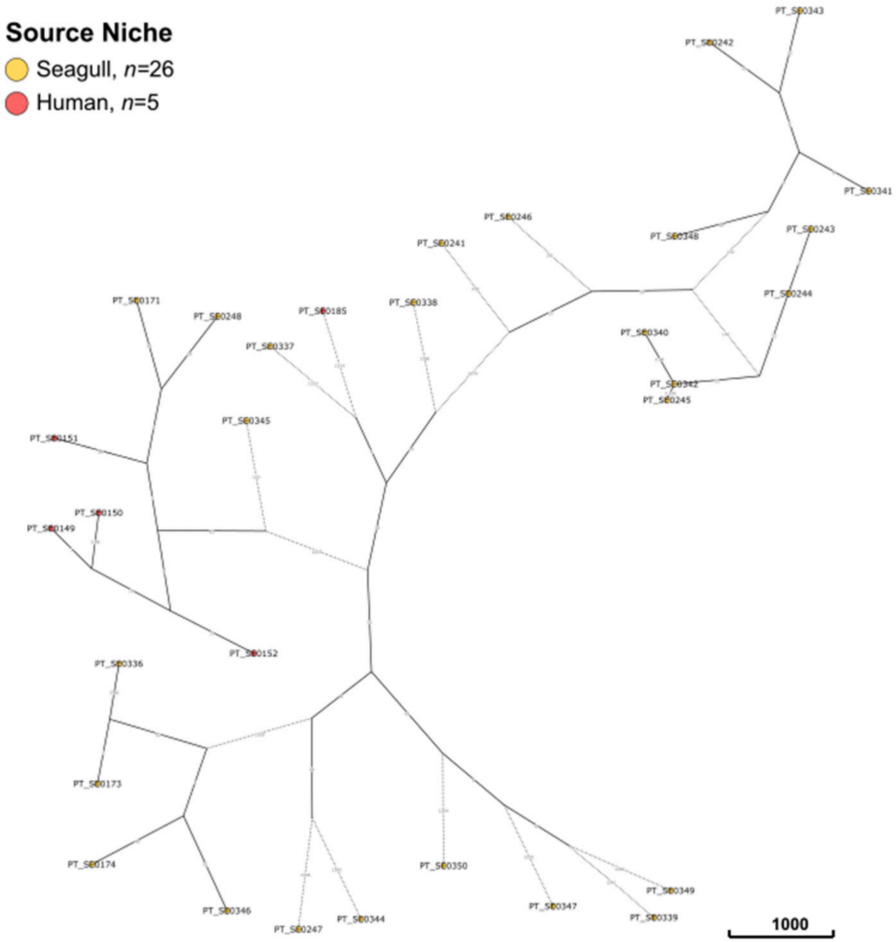

Figure S2. Comparative GrapeTree analysis of the 2008 *Salmonella* isolates from gulls examined in this study and from Portuguese human clinical isolates available in Enterobase. The core genome minimum spanning tree was created within the Enterobase pipeline using the NINJA NJ algorithm and GrapeTree tool. The scale bar corresponds to the number of cgMLST allelic differences.

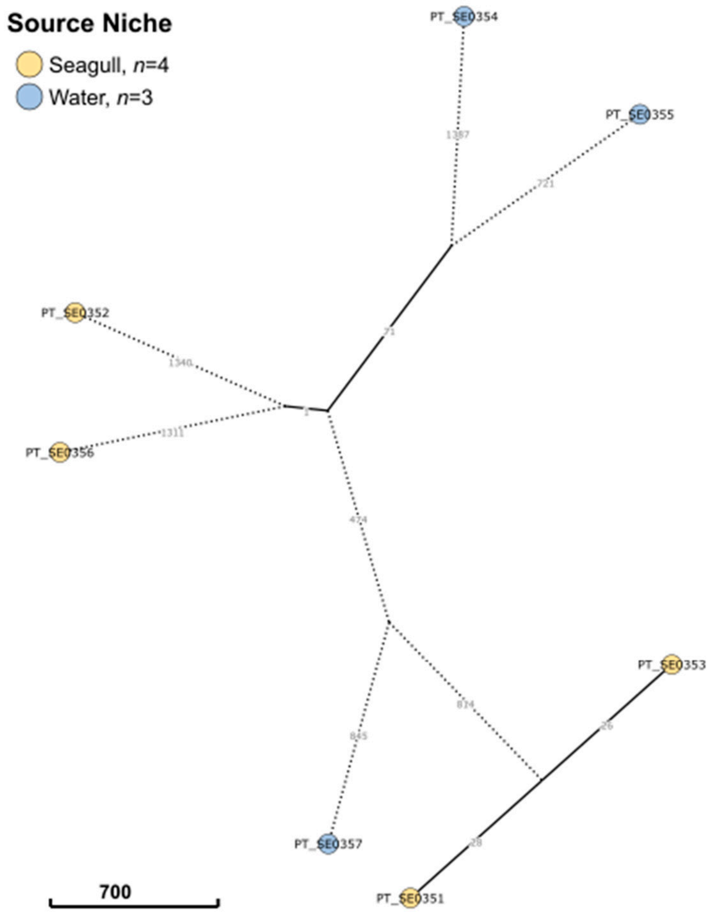

Figure S3. Comparative GrapeTree analysis of the 2023 *Salmonella* isolates from gulls and water examined in this study. The core genome minimum spanning tree was created within the Enterobase pipeline using the NINJA NJ algorithm and GrapeTree tool. The scale bar corresponds to the number of cgMLST allelic differences.
